# Proteotranscriptomic Profiling of the Toxic Mucus of *Kulikovia alborostrata* (Pilidiophora, Nemertea)

**DOI:** 10.3390/toxins17010005

**Published:** 2024-12-26

**Authors:** Vasiliy G. Kuznetsov, Daria I. Melnikova, Sergey V. Shabelnikov, Timur Yu. Magarlamov

**Affiliations:** 1 A.V. Zhirmunsky National Scientific Center of Marine Biology, Far Eastern Branch, Russian Academy of Sciences, 690041 Vladivostok, Russia; 2 Institute of Cytology, Russian Academy of Sciences, 194064 St. Petersburg, Russia

**Keywords:** *Kulikovia alborostrata*, mucus, secretions, toxins, cysteine-rich peptides, proteomics, transcriptomics, RT-qPCR, nemertea

## Abstract

Nemertea is a phylum of bilaterally symmetrical, coelomate, unsegmented worms, also known as ribbon worms. Most species of the phylum Nemertea are marine predators that contain toxins in the single-celled glands of the proboscis and/or integument. Recent transcriptomic studies have shown that nemerteans from all taxonomic groups possess a wide range of putative protein and peptide toxins, while the proteomic data for these animals are highly limited. In this study, proteotranscriptomic analysis was used to investigate the major protein components of the poison of the nemertean *Kulikovia alborostrata*. We identified 146 transcripts of putative toxins in the transcriptome of *K*. *alborostrata* and five putative toxins among the secreted proteins and peptides of the mucus of the animal. The expression levels of cysteine-rich peptides found in the mucus with similarity to known toxins were evaluated in different parts of the body of the worm by quantitative real-time PCR. The high level of expression of investigated peptides in the integument indicate the protective function of these toxins. Overall, this supports the idea that the mucus of nemerteans is a valuable source of peptide and protein toxins.

## 1. Introduction

The development of modern approaches for joint proteomic and transcriptomic analysis significantly changed the level of the studies of toxin-bearing animals [1]. A comprehensive characterization of proteinaceous toxins in animals that do not possess anatomically distinct venom glands became possible. Nemerteans are one such animal [2].

The phylum Nemertea is comprised of predominantly marine unsegmented worms and is currently divided into three phylogenetic groups: Palaeonemertea, Pilidiophora (Hubrechtiiformes and Heteronemertea), and Hoplonemertea [3,4]. Most nemerteans, except for a small number of symbiotic species, are predators [5,6,7,8,9]. They spend most of their time sitting in algal rhizoids or under rocks and waiting for prey [10] or very slowly moving along the bottom in search of food [11,12,13,14,15,16]. Despite their slowness and soft muscular body, nemerteans are rarely preyed upon by typical predators such as fish and decapod crabs [17,18,19,20,21,22]. Distinctive features of nemerteans are the abundant mucous secretion on the surface of the body, which is responsible for the defense of the animal, and an eversible proboscis apparatus, which aids in prey capture [5]. Despite nemerteans not having distinct poisonous glands, they contain toxins in the glandular cells of the proboscis and integument epithelium, which provide more efficient predation and defense [23,24,25].

While the proboscis is everted, the glandular epithelium takes an external position and directly contacts with the prey [7,26]. Glandular cells of the proboscis epithelium secrete a sticky toxic mucus that contains various types of secretion products that may serve immobilization and adhesion of prey to the proboscis [23,27,28]. In palaeonemerteans and pilidiophorans, toxin-containing glands are distributed evenly throughout the epithelium of the proboscis [9,23,24,25,29,30]. In hoplonemerteans, toxin-containing glands are evenly scattered throughout the anterior part of the proboscis, while the secretion accumulates in large quantities in the middle (stylet bulb) and posterior parts of the proboscis [16,23,31]. Nemerteans possess strategies allowing penetration of toxins into the victim’s body [32]. The proboscis of palaeonemerteans and pilidiophorans, with the exception of species of the genera Baseodiscus [33] and Sonnenemertes [34] and some species of Hubrechtella [35], bears special granules, called pseudocnidae, enclosing a hollow thread-like tubule. The thread located inside pseudocnidae, by analogy with the cnidae of Cnidarians, is capable of everting outwards [27,36,37] and can be ejected into the integument of prey [27,32,38]. Hoplonemerteans pierce the integument of prey with a calcified spine (stylet) located in the middle part of the proboscis [7,26]. Secretions from the stylet bulb and posterior part of proboscis are injected into the prey [23,32,39].

The glandular cells, participating in mucus production, are evenly distributed in the integument of all nemerteans [40]. The abundant mucous layer on the surface of the animal acts both as a lubricant, providing mechanical protection against physical damage [40], and a repellent, protecting against potential predators [41,42,43]. A number of studies have demonstrated that in response to mechanical or electrical stimulation [44,45], as well as an attack of a potential predator in the laboratory [5], nemerteans quickly release high amounts of mucus. It is considered that neurotoxins, found in the mucus of many nemerteans, are responsible for deterring predators [2,41,46,47]. Moreover, not all glandular cells located in the integument participate in the excretion of toxins into the mucus. Specialized toxin-producing cells were discovered in the integument of several species of nemerteans [24,25,29].

Numerous toxins have been found among nemerteans of all three phylogenetic groups [2,46]. The entire spectrum of toxins derived from nemerteans may be divided into three main groups: pyridine alkaloids, tetrodotoxin and its analogs (TTXs), and peptides and proteins [2]. And if pyridine alkaloids are found only in hoplonemerteans [28,31,39,48,49,50], and TTXs only reach significant concentrations in palaeonemerteans and are found in other nemertean groups in trace amounts [51,52], peptide and protein toxins are present in most nemerteans studied [2,53,54]. Recent molecular studies identified in the transcriptomes of different nemerteans from 3 to 200 transcripts encoding putative toxic peptides and proteins [53,54,55,56,57]. The largest number of putative toxins has been observed in transcriptomes of nemerteans from the Pilidiophora group [54,56]. The expansion of toxin genes may result from an increased degree of gene duplication in this nemertean group, causing redundant proteins and their neofunctionalization [56]. Most currently described nemertean-specific toxins were isolated from pilidiophoran species and include cytolysins A II-IV [42], neurotoxins B I-IV [58,59], α- and β-nemertides [60], and parborlysin [61]. Besides the nemertean species in which they were originally discovered, these cysteine-rich peptides (CRPs) and their homologues were found in other representatives of Pilidiophora [53,54,55,56]. Moreover, transcriptomic studies of nemerteans from all taxonomic groups revealed a variety of secreted CRPs, containing inhibitor cystine knot (ICK) and stichodactyla-like domains, Alpha-KTx-Like/Beta-Defensin-Like/Myticin-like peptides, and conotoxin-like proteins, which may potentially perform toxic activity [54]. However, the diversity of toxic CRPs in nemerteans may be much higher, since high genetic variability in these peptides does not always allow their identification through databases [62].

In this study, we investigated the protein and peptide toxin content in the transcriptome of the whole body and in the mucus of the pilidiophoran *Kulikovia alborostrata*, a nemertean species widely spread off the coasts of the Sea of Japan and the Yellow Sea, using a combined transcriptomic and proteomic approach. The expression levels of the selected CRPs with a putative toxic activity were evaluated in different parts of the body of the worm by quantitative real-time PCR (qRT-PCR). A distinctive feature of *K*. *alborostrata*, as well as other heteronemerteans, from other nemertean groups is the presence of an additional subepidermal glandular layer, called cutis [40]. The presence of additional glandular structures, resulting in the producing of high amounts of mucus, allowed heteronemerteans to successfully colonize a wide range of habitats [40]. Recent studies showed that cutis glands contain a variety of components, including proteinaceous [60] and non-proteinaceous toxins [29,63].

## 2. Results

### 2.1. Transcriptome of K. alborostrata

The transcriptome of the nemertean *K. alborostrata* was obtained using a hybrid assembly, generated from Oxford Nanopore and Illumina reads. RNA for the transcriptome was extracted from the cross-sections, obtained from the precerebral region of the body of *K*. *alborostrata* (before the mouth). Illumina sequencing generated 141.8 million raw sequence reads with a maximum read length of 101 bp. After the removal of low-quality reads, 79.8 million clean reads were obtained. Two Nanopore sequencing runs yielded in 5.2 million sequence reads with an average read length of 1185 bp. The de novo assembly produced 383,778 contigs with an average and maximum read lengths of 1439.9 bp and 13,756 bp, respectively. Benchmarking Universal Single-Copy Orthologs (BUSCO) (https://gitlab.com/ezlab/busco, accessed on 20 October 2021) analysis of the *K. alborostrata* transcriptome’s completeness indicated that 860 (90.1%) of 954 core single-copy metazoan genes were present in the assembly, with 795 (83.3%) complete and 65 (6.8%) fragmented genes. Protein prediction using TransDecoder (https://github.com/TransDecoder/TransDecoder, accessed on 20 October 2021) resulted in 126,932 sequences longer than 70 amino acids, 54,090 (41.6%) of which were annotated using BLASTP (https://blast.ncbi.nlm.nih.gov/Blast.cgi, accessed on 8 April 2024) homology search (best match only, E-value ≤ 10^−5^) against SwissProt database (Uniprot.org, accessed on 8 April 2024) (Supplementary File S1).

### 2.2. Toxins Identified in the Transcriptome of K. alborostrata

**Figure 1:**
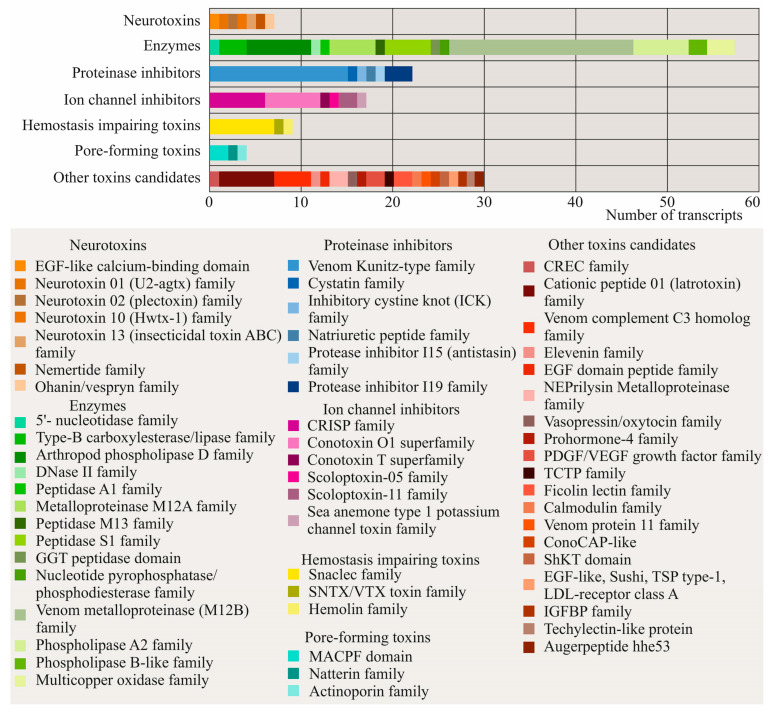
Putative toxin families/domains identified in the transcriptome of *Kulikovia alborostrata*. The figure illustrates the proportional distribution of toxin family’s/domain’s transcripts in the transcriptome.

Putative toxins in the transcriptome of *K. alborostrata* were identified by blasting the SwissProt, ToxProt, and nemertean toxins databases (E-value ≤ 10^−5^) (Supplementary File S2). The protein products of 146 coding transcripts were predicted to have toxic activity (Supplementary Table S1). Identified transcripts corresponded to seven toxin groups according to their predicted functions and were classified into 56 gene families based on their cysteine scaffolds and amino acid sequences (Figure 1). Enzymes, which included 57 transcripts, constituted the amplest group in the transcriptome. Most transcripts annotated as enzymes were encoded metalloproteases, phospholipases, and peptidases. Other enzyme coding sequences were assigned to multicopper oxidases, carboxylesterases, acetylcholinesterase, phosphodiesterase, 5′-nucleotidase, and DNase II-like protein. The enzyme group was followed by a group of protease inhibitors, which accounted for 22 transcripts. The majority of sequences were serine protease inhibitors. The rest of the protease inhibitor coding sequences were annotated as trypsin, prophenoloxidase, metalloprotease inhibitors, cystatin, and knottin. Seventeen coding sequences had an identity with ion channel blockers. Among this group, CRPs, including allergens, conotoxins, scoloptoxins, and actitoxin-like protein were found. Nine transcripts corresponded to hemostasis impairing toxins and included C-type lectin-like proteins, neoverrucotoxin, and hemolin. Seven transcripts related to neurotoxic activity were assigned to theraphotoxins, nemetoxins, glycerotoxin, agatoxin, ctenitoxin, vespryn, and nemertide alpha-1. Four transcripts, identified as pore-forming toxins, corresponded to perivitellin, natterin, and tereporin. Finally, 30 transcripts, were classified as other toxin candidates. Polypeptides of this group were discovered in the venom of other animals and their biological function, or molecular target, had not been demonstrated experimentally. Among this group, two transcripts were attributed to unassigned peptides, as a particular family or domain could not be identified.

### 2.3. Toxins Identified in the Mucus Proteome of K. alborostrata

**Figure 2:**
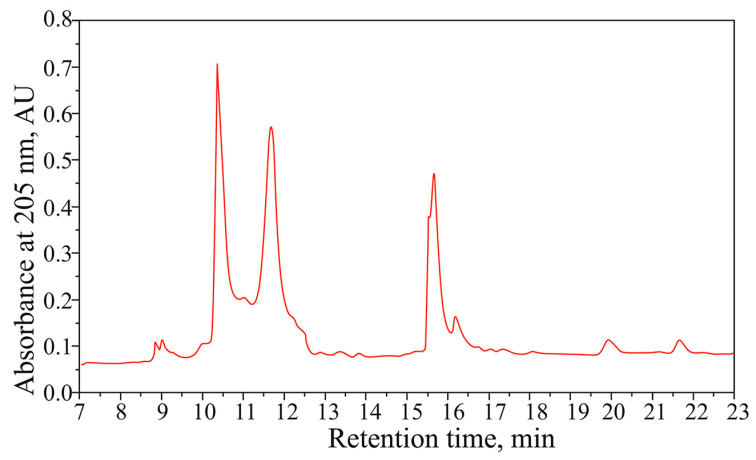
Reversed-phase high-performance liquid chromatography plot of the mucus sample of *Kulikovia alborostrata*.

For proteomic analysis, mucus samples of nine specimens of *K*. *alborostrata* were used. Preliminary assessment of the mucus composition was performed using reversed-phase high-performance liquid chromatography (RP-HPLC). Up to 19 peaks were resolved in *K*. *alborostrata* mucus samples (Figure 2). Total peak area varied from 58.4 to 152.5 AU*μL, yielding mean total protein concentration of 30.0 ± 16.7 μg/mL (mean ± sd).

Proteomic analysis was performed on the mucus protein extracts of *K. alborostrata*, with a total of nine independent runs in a matrix-assisted laser desorption/ionization time-of-flight/time-of-flight (MALDI-TOF/TOF) mass spectrometer. Using transcriptomic data obtained in the current study, a total of 30 proteins and peptides were identified in the mucus samples, of which 19 had signal peptide sequences (Supplementary Table S2). These included 12 proteins and seven peptides (sequences < 100 amino acids in length), of which five sequences had significant similarities in the UniProt and ToxProt databases to known toxins (Table 1, Supplementary File S3).

Among secreted peptides, nemertide alpha-1 (ORF|076246), U1-nemetoxin-Csp1a (ORF|006024), and four unknown peptides (ORF|001200, ORF|005232, ORF|084558, and ORF|096413) containing six and more cysteine residues in their sequences were picked for further analysis with ConoServer (https://www.conoserver.org/, accessed on 8 April 2024). A 77-residue peptide under the identifier ORF|001200, along with nemertide alpha-1 and U1-nemetoxin-Csp1a, were classified into cysteine framework VI/VII (C-C-CC-C-C). Two 65-(ORF|084558) and 69-(ORF|096413) residue peptides belonged to a cysteine framework IX (C-C-C-C-C-C-C). A 72-residue peptide under the identifier ORF|005232 was classified into a cysteine framework XXII (C-C-C-C-C-C-C-C-C). Phyre2-based tertiary structure modeling generated high-confidence predictions (>80%) for peptides from cysteine framework VI/VII (Supplementary File S4). A 3D model of the peptide under the identifier ORF|076246 confirmed its alignment with nemertide alpha-1 toxin with a confidence level of 94.5% and 50% coverage. The ORF|006024 peptide shared 50% identity with beta-theraphotoxin-ps1a toxin with a confidence level of 98%. Not identified with proteotranscriptomic analysis, the peptide under the identifier ORF|001200 had 61% identity with the antimicrobial protein kalata-b1 with a confidence level of 81.2%. The 3D model alignments of peptides from the other cysteine frameworks had confidence levels that were too low (<30%) to include them in further analysis. CSPred functional analysis (https://bitbucket.org/sm_islam/cystine-stabilized-proteins/src/master/, accessed on 3 June 2024) classified peptides from cysteine framework VI/VII as ion channel blockers with a probability score > 0.8 (Table 2). With a lower probability, peptides under identifiers ORF|001200 and ORF|076246 were assigned to the antimicrobial (*p* = 0.45) and serine protease inhibitor (*p* = 0.34) groups, respectively.

**Figure 3:**
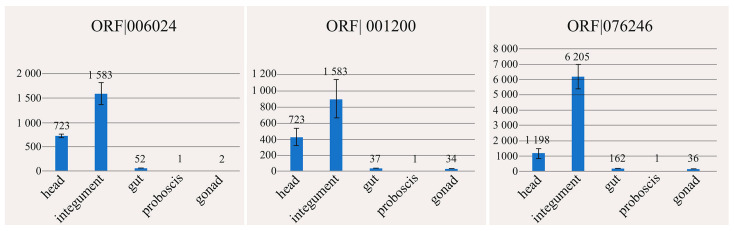
Relative expression levels of three putative toxins identified in the mucus proteome of *Kulikovia alborostrata* in different parts of the body of the worm. Gene expression levels were quantified by quantitative real-time PCR using the 2^−∆∆Ct^ method. Data represent the mean of three independent replicates ± SEM. Reference gene: 60S ribosomal protein L32. Calibrator sample: proboscis.

Gene expression for three putative toxic peptides (ORF|076246, ORF|006024, ORF|001200) was evaluated in the head, integument, proboscis, intestines, and gonads of *K. alborostrata* by qRT-PCR (Figure 3). The proboscis was used as calibrator. A significantly higher transcription level in the head and integument was observed for all the selected genes. The highest transcription level (in two of three biological replicates) was observed for nemertide alpha-1 toxin (ORF|076246).

## 3. Discussion

**Figure 4:**
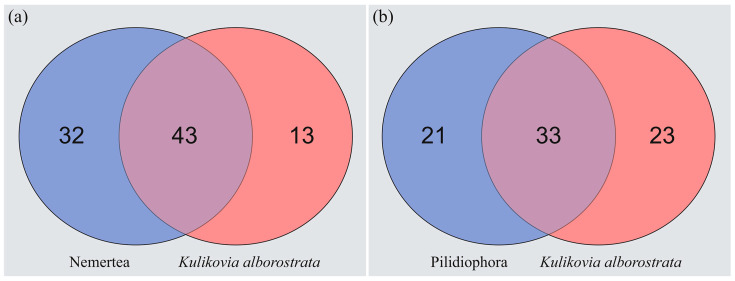
Venn diagram showing the number of putative toxin gene families shared between *Kulikovia alborostrata* and all nemerteans (**a**) and *K*. *alborostrata* and pilidiophorans (**b**).

Recent transcriptomic studies of nemerteans belonging to different taxonomic groups have shown that these animals possess a wide range of putative protein and peptide toxins [54,55,56]. In the current study, the transcriptome of the pilidiophoran *K. alborostrata* is reported for the first time. We identified 146 transcripts of putative toxins and other toxin candidates and categorized them into 56 toxin gene families. In previous studies, a total of 75 toxin gene families were identified in the transcriptomes of nemerteans, of which 54 were found in pilidiophorans (Supplementary Table S3) [53,54,55,56,58,59,60,64]. Comparative analysis of toxin gene families in transcriptomes of nemerteans revealed that *K. alborostrata* share 43 families with all nemerteans (Figure 4a), and 33 families with other pilidiophorans (Figure 4b). According to the number of common toxin gene families in transcriptomes, *K. alborostrata* is close to the pilidiophorans *Lineus longissimus* and *Lineus sanguineus* (20 common families) and palaeonemertean *Cephalothrix* cf. *simula* (22 common families) (Supplementary Table S3). These data are consistent with the previous large-scale study of nemertean transcriptomes, which revealed that representatives of Pilidiophora and Palaeonemertea classes share more toxin gene families compared to hoplonemerteans [54]. Several authors associate differences in toxin number and diversity between nemertean classes with the different diet preferences [54,55]. Thus, most palaeonemerteans and pilidiophorans possess a wide range of prey, while hoplonemerteans have a more specialized diet [16,27,47,65].

The proteomic approach combined with the transcriptomic data was used to analyze the composition of mucus of *K. alborostrata*. Out of 19 secreted proteins and peptides found in the mucus, only 7 were annotated (Supplementary Table S2). In previous studies revealing the protein composition of the mucus of nemerteans, a large proportion of secreted mucus proteins and peptides were also not annotated [55,56,64]. A large number of unidentified protein components found both in mucus and in other parts of the body of nemerteans indicates a significant gap in our understanding of the molecular biology of these animals and opens new perspectives in the search for novel toxins.

In the current study, five putative toxins were identified in the mucus of *K. alborostrata*. Most of the toxins found have been previously described in the mucus and body proteomes of some hoplonemerteans and pilidiophorans. Thus, astacin-like metalloproteinases have been found in the mucus proteome of the hoplonemertean *Amphiporus lactifloreus* [64], in the body and mucus proteomes of the hoplonemertean *Nemertopsis pamelaroeae* [56], and in the body proteome of the pilidiophoran *L. sanguineus* [56]. U-actitoxin-like, named U-nemertotoxin-2, and antistasin-like protease inhibitors were described in the mucus proteomes of *A. lactifloreus* [64] and *L. sanguineus* [56], respectively. Antistasin-like toxin has been also found in the proboscis proteome of the hoplonemertean *Antarctonemertes valida* [55]. Nemertide alpha, being one of the most studied nemertean-specific peptide toxins, was first discovered and isolated from the mucus of the pilidiophoran *L. longissimus* [60], and further found in the transcriptomes of many species of the Lineidae family [56]. In a recent study, nemertide alpha was described in the mucus proteome of the Antarctic nemertean *Parborlasia corrugatus* [57]. A peptide with significant similarity to U1-nemetoxin-Csp1a, an insect-specific neurotoxic peptide isolated from the venom of a spider from the genus *Calisoga*, is discovered in nemerteans in the current study for the first time. Interestingly, cytotoxin-A [66] and its homologue parborlysin [57,67], discovered in the mucus of pilidiophorans and quite specific to this group of nemerteans [53,54,56,64], were not found in either transcriptome or mucus proteome of *K. alborostrata*.

A mixture of toxins with various physiological effects contained in the mucus of *K*. *alborostrata* may act as a deterrent agent against protentional predators. Thus, toxins with anticoagulant (ORF|016168), lysing (ORF|006949), and hemolytic (ORF|006816) activities may damage the integument of a potential predator in the case of an attack on the nemertean, while neurotoxins (ORF|076246, ORF|006024), penetrating through the wound, directly act on various targets of the nervous system, exerting a paralytic effect. The possibility of a synergistic effect of mucus toxins aimed at repelling predators has been suggested for other nemerteans [55,57,64]. However, further studies are needed to verify the accuracy of proteo-transcriptomic annotations with experiments on isolated proteinaceous toxins.

Few proteomic studies conducted on nemerteans show that the number of annotated toxins in the body and mucus are many fewer than in the transcriptomes of the same nemertean species [55,56,64]. It is possible that the majority of peptide and protein toxins in nemerteans is concentrated in the proboscis, where the toxic mixture containing various components, including proteases, phospholipases, protease inhibitors, pore-forming toxins, and neurotoxins, provides effective penetration of the toxin in the victim`s body. Thus, many of the toxins identified in the transcriptome, but absent in the mucus proteome, of *K. alborostrata*, including conotoxin-like and plancitoxin-like peptides, calglandulin, phospholipase A2, neuropeptide prohormone-4, 5′- nucleotidase, and proteins related to insulin like growth factor and binding protein family, are found in the transcriptomes of the proboscis of hoplonemerteans *A. valida* and *A. lactifloreus* [55,64]. Verdes and colleagues showed that some of the above-mentioned toxins were found only in the proboscis [55]. A relatively small variety of toxins found in the proteomes of the integument and mucus of nemerteans can be explained by the broad spectrum of their alleged action. For example, alpha-nemertides, extensively found in the mucus and skin of lineid nemerteans, possess high affinity and selectivity to voltage-gated sodium channels and are found to be toxic to a wide range of arthropods [68]. Other pilidiophoran-specific toxins found in the skin and mucus, A-cytolysin and parborlysins, are known for cytolytic and hemolytic activities [2].

If a gene of an unknown polypeptide found in the proteo-transcriptomic study exhibits a high level of expression in the specialized poison glands of an animal, it may be indicative of a toxic function of this polypeptide. However, nemerteans do not have distinct poisonous anatomical structures, and their single-celled glands secreting toxins are located in the proboscis and/or integument epithelium [23,24,25]. In the current study, the expression levels of the CRPs, annotated as nemertide alpha-1 (ORF|076246) and U1-nemetoxin-Csp1a (ORF|006024), and an unknown CRPs with a 61% identity with the antimicrobial protein kalata-b1 (ORF|001200), were evaluated in the head, integument, proboscis, intestines, and gonads of *K. alborostrata*. Expression of all three peptides was significantly higher in the head and integument compared with the other parts of the body, with the highest level was in the integument (Figure 3). The results obtained suggest that the primary function of these putative toxins is defensive. Similar results were obtained in the study of Jacobsson and colleagues [60] on *L. longissimus*. The distribution of nemertide alpha-1, alpha-2, and beta-2 were analyzed using matrix-assisted laser desorption ionization mass spectrometry. The analyzed toxins were located in the epidermis and mucus layer of the worm.

## 4. Conclusions

The integrated transcriptomic and proteomic approach used in the current study provides an overview of the major protein components of the *K. alborostrata* poison. Out of the 146 putative protein toxins annotated in the transcriptome of *K. alborostrata*, five putative toxins, including two CRPs, were secreted in the mucus. Among unannotated mucus peptides, four more CRPs containing ICK motifs were revealed. Peptides containing an ICK motif are thought to have a high structural stability and functional diversity and are frequently found in the venom of well-studied poisonous animals, like scorpions, spiders, and aquatic cone snails. A broad spectrum of biological activities combined with the high resistance of ICK peptides makes them a prospective object for pharmacological and bio-engineering studies. Despite little interest in nemertean toxicology, our study and previous studies demonstrated that the mucus of pilidiophoran nemerteans is a valuable source of novel peptide and protein toxins, specifically ICK peptides.

## 5. Materials and Methods

### 5.1. Nemerteans Collection

**Figure 5:**
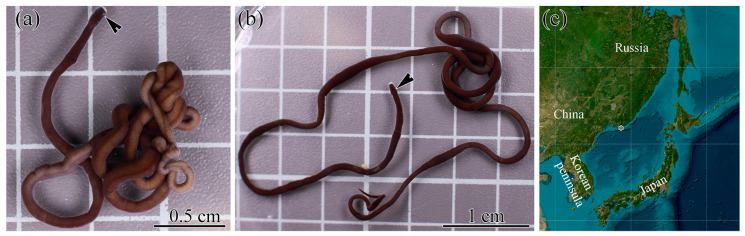
*Kulikovia alborostrata* (Takakura, 1898) live specimens and collection site. (**a**) Female, (**b**) male, (**c**) collection site (asterisk). The black arrowheads point to the head region of worms.

The specimens of *Kulikovia alborostrata* (Takakura, 1898) were collected in rhizoids of the brown algae Saccharina sp. at a depth of 0.5–1.5 m at Spokoynaya Bay (42.7090 N, 133.1809 E), Sea of Japan (Figure 5). The specimens were collected during four summer seasons in 2019, 2020, 2023, and 2024. Algal rhizoids were manually cut off the rocks, carried to the Vostok Marine Biological Station of the A.V. Zhirmunsky National Scientific Center of Marine Biology, Far Eastern Branch, Russian Academy of Sciences (Vladivostok, Russia) in tanks filled with the local seawater, and replaced in tanks with aerated seawater at 17–20 °C until nemerteans came out of them. Collected nemerteans were kept individually in aerated aquaria with seawater sterilized with a 0.45 μm MF-Millipore™ membrane filter (Merck Millipore, Burlington, MA, USA) at 17 °C without feeding.

**Figure 6:**
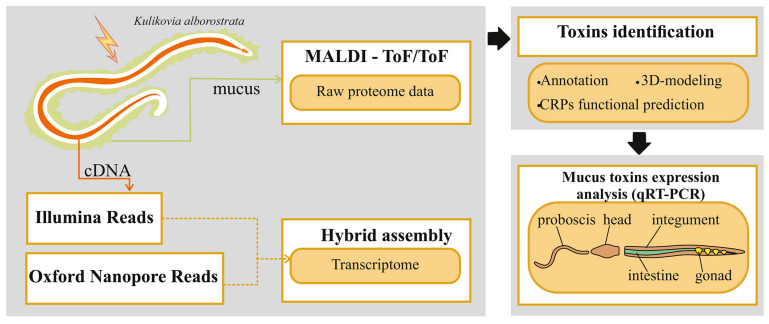
Schematic diagram of the experiment design.

Collected animals were used for further transcriptomic and proteomic studies (Figure 6).

### 5.2. Taxonomic Assignment

Nemertean specimens were identified based on their morphology and the partial sequences of mitochondrial cytochrome c oxidase subunit I (COI) gene. Total DNA from individual specimens was extracted using the E.Z.N.A. Mollusc DNA Kit (Omega Bio-tek, Norcross, GA, USA) in accordance with the manufacturer’s protocol. PCR amplification of the 710 bp COI gene fragment was performed using LCO1490 (5′-GGTCAACAAATCATAAAGATATTGG-3′) and HCO2198 (5′-TAAACTTCAGGGTGACCAAAAAATCA-3′) primer pair [69]. PCR reactions contained 1 μL of each primer (10 μM), 1 μL of template DNA, and 10 μL of GoTaq^®^ Green Master Mix (Promega, Madison, WI, USA) in a total volume of 20 μL. PCR program included an initial denaturation step of 3 min at 94 °C, followed by 45 cycles comprising 30 s denaturation at 94 °C, 25 s annealing at 51 °C, and 90 s elongation at 72 °C, and a final extension step of 5 min at 72 °C. The amplified products were purified using QIAquick PCR Purification Kit (Qiagen, Hilden, Germany) and sequenced using a BigDye Terminator Cycle Sequencing Kit (ver. 3.1, Applied Biosystems, Waltham, MA, USA) and the same PCR primers on an ABI Prism 3500 Genetic Analyzer (Applied Biosystems, Waltham, MA, USA). Sequence data were proofread using Chromas Lite software, v. 2.6.6 (Technelysium Ltd., Brisbane, Australia) (https://technelysium.com.au/wp/chromas/, accessed on 1 September 2021). Sequence fragments were merged into consensus sequences and aligned using MEGA7 (Molecular Evolutionary Genetics Analysis) software, v. 7.0 (https://www.megasoftware.net/, accessed on 1 September 2021) [70]. Taxonomy of the specimens was identified using BLAST [71].

### 5.3. Transcriptomic

#### 5.3.1. RNA Extraction, cDNA Synthesis, and Sequencing

For total RNA extraction, 15-25 mg cross-sections from two *K. alborostrata* samples were dissolved in RNALater solution (Thermo Fisher Scientific, Waltham, MA, USA). Cross-sections were obtained from the precerebral region of the body of worms (before mouth). RNA was extracted using TRIzol reagent (Thermo Fisher Scientific, Waltham, MA, USA) in accordance with the manufacturer’s instructions. Total RNA was quality-controlled and quantified using a spectrophotometer BioSpec-nano (Shimadzu, Kyoto, Japan). The extraction of mRNA from total RNA was performed using the NEBNext Poly(A) mRNA Magnetic Isolation Module kit (New England Biolabs, Ipswich, MA, USA). The cDNA was synthesized using the MINT2 kit (Eurogen, Moscow, Russia), purified with the Bioline ISOLATE II PCR and Gel Kit (Meridian Bioscience Inc., Cincinnati, OH, USA) and normalized using the Trimmer-2 kit (Eurogen, Moscow, Russia). The amplification of normalized cDNA was performed with the use of Encyclo polymerase and M1 primer (5′-AAGCAGTGGTATCAACGCAGAGT-3′) from the MINT2 kit. The concentration and purity of cDNA were assessed using a Qubit 4 fluorometer (Thermo Fisher Scientific, Waltham, MA, USA). The cDNA was sequenced by a combination of long- and short-read methods on a MinION Mk1B sequencer (Oxford Nanopore Technologies, Oxford, UK) and an Illumina NovaSeq6000 platform (Illumina, San Diego, CA, USA) (Figure 6). Library for nanopore sequencing was prepared using Direct cDNA Sequencing Kit SQK-DCS109 (Oxford Nanopore Technologies, Oxford, UK) according to the manufacturer protocol. Two Nanopore sequencing runs were performed on R9.4 and R.10.3 flow cells using standard software MinKNOW v3.6.0. Libraries for Illumina sequencing were prepared using the NEBNext Ultra II FS DNA Library Prep Kit for Illumina (New England Biolabs, Ipswich, MA, USA) following the manufacturer’s guidelines for the samples with cDNA concentration > 100 ng. AMPure XP reagent (Beckman Coulter, Brea, CA, USA) was used to clean up the prepared library. Illumina sequencing was performed at The Center of Genetics and Reproductive Medicine “Genetico” (Moscow, Russia).

#### 5.3.2. Transcriptome Assembly and Annotation

Oxford Nanopore reads were subjected to Porechop v. 0.2.4 (https://github.com/rrwick/Porechop, accessed on 1 October 2021) to remove adapters and chimeric sequences. Raw Illumina reads were quality-control-checked using FastQC v. 11.9 (https://www.bioinformatics.babraham.ac.uk/projects/fastqc/, accessed on 5 October 2021). Adapters and low-quality Illumina reads were trimmed using Trimmomatic v. 0.39 [72] with the addition of the used adapter sequences to the database and following settings: LEADING:20, TRAILING:20, SLIDINGWINDOW:4:15, MINLEN:50, HEADCROP:10. The resultant quality-trimmed Illumina reads were used for ten rounds of Oxford Nanopore read correction using minimap2 v. 2.24-r1122 (https://github.com/lh3/minimap2, accessed on 10 October 2021) [73] and racon v. 1.4.13 (https://github.com/isovic/racon, accessed on 10 October 2021). High-similarity sequences (≥97%) were removed from Oxford Nanopore reads using CD-HIT v. 4.8.1 (https://github.com/weizhongli/cdhit, accessed on 15 October 2021) [74]. The trimmed Illumina reads were de novo assembled into contigs by SPAdes v.3.15.3 (https://github.com/ablab/spades, accessed on 15 October 2021) [75]. The hybrid assembly of merged contigs from Illumina reads and the corrected Oxford Nanopore reads was performed using minimap2 v. 2.24-r1122 and racon v. 1.4.13. The completeness of the transcriptome annotation was assessed with BUSCO v. 5.2.1 [76]. Possible open reading frames (ORFs), encoding 70 amino acids or more, and their translated protein sequences were obtained using TransDecoder v. 5.5.0 (https://github.com/sghignone/TransDecoder, 20 October 2021). Functional annotation of the transcriptome was performed using InterProScan [77]. Putative toxic peptides and proteins were identified using BLASTP homology search (best match only, E-value ≤ 10^−5^) against three databases: SwissProt with 571,407 entries, ToxProt with 7890 entries, and nemertean toxins database with 64 entries.

Raw transcriptomic data are available at NCBI’s GenBank under Bioproject PRJNA1190494 (accession numbers SRR31507531, SRR31507530, SRR31507529, SRR31507528).

### 5.4. Proteomics

#### 5.4.1. Mucus Collection and Protein Extraction

Mucus samples from nine specimens of *K. alborostrata* were collected using electrical stimulation according to Vlasenko et al. [45]. Individual specimens were placed in Petri dishes with 1 mL of sterile seawater. Mucus secretion was promoted by a short electric current pulse at 6 V, 2 s duration, induced with copper electrodes placed in the water. Individual mucus samples were collected in sterile 15 mL tubes and ultrasonicated using a Sonopuls HD 2070 homogenizer (Bandelin Sonopuls, Berlin, Germany) for 40 s (at a frequency of 20 kHz; amplitude, 228 μm; working cycle, 0.8 s; and interval, 0.2 s). The homogenates were centrifuged at 8300× *g* for 30 min at 4 °C, and the supernatants were purified by Oasis^®^ MCX solid-phase extraction cartridges (Waters, Milford, MA, USA). The cartridge was activated by 100% methanol and equilibrated by 2% aqueous formic acid. The supernatant acidified with 2% aqueous formic acid was loaded onto the cartridge, washed with 0.1 N HCl and 100% methanol successively, and eluted with 5% NH4OH in acetonitrile. The eluates were dried in a rotor vacuum evaporator (Labconco, Kansas City, MO, USA) and kept at −20 °C until used.

#### 5.4.2. RP-HPLC Analysis of Mucus

Dry mucus samples were dissolved in 0.5% formic acid and analyzed on a micro-bore HPLC system (MiLiChrom A-02, EcoNova, Novosibirsk, Russia). A sample volume of 100 μL was loaded onto a Jupiter C5 reversed-phase column (2 mm × 100 mm, 5 μm, 300 Å, Phenomenex, CA, USA) and separated using a linear gradient of 0–100% B over 23 min at a flow rate of 200 μL·min^−1^. The mobile phases used were A, 5% (*v*/*v*) acetonitrile, 0.1% (*v*/*v*) TFA in water and B, 50% (*v*/*v*) acetonitrile, 0.1% (*v*/*v*) TFA in water. Absorbance was monitored at 205 nm with UV detector slit width of 2 nm. Total protein concentration was estimated from the total peak area using A205 nm = 31 mL mg^−1^ cm^−1^ [78].

#### 5.4.3. HPLC-MALDI-TOF-TOF-MS/MS Analysis

Dry mucus samples were dissolved in 8 M urea, followed by treatment with dithiothreitol 10 mM for 15 min at 80 °C. Alkylation was performed by iodoacetamide 20 mM for 30 min at room temperature. Then, proteins were digested with trypsin using a 1:50 ratio of protease to substrate (*w*/*w*) (Trypsin Gold, Promega, Madison, WI, USA) overnight at 37 °C, acidified with formic acid, and desalted using 100 mg Strata-X solid-phase extraction cartridges (Phenomenex, Torrance, CA, USA) with the recommended washing buffers. Proteins eluted in 2% formic acid in methanol/acetonitrile (1:1) were dried in a rotor vacuum evaporator, resuspended in 50 μL of 1% aqueous formic acid, and filtered through 0.22 μm polyvinylidene fluoride filter (Sigma-Aldrich, St. Louis, MO, USA).

RP-HPLC was performed on a NanoLC-Ultra 2Dplus system (SCIEX, Framingham, MA, USA) using a 0.1 × 100 mm Chromolith CapRod RP-18e HR reversed-phase column (Merck Millipore, Burlington, MA, USA) at a flow rate of 400 nL/min. Mobile phases consisted of 0.2% aqueous trifluoroacetic acid (TFA) (A) and 80% aqueous acetonitrile (B). The column was operated at a room temperature of 22–24 °C. The effluent from the column was mixed with a matrix solution, containing 4 mg/mL α-cyano-4-hydroxycinnamic acid in 95% methanol with 0.1% TFA, and two calibration standards, bradykinin 2–9 (30 pM/mL) and ACTH 18–39 (60 pM/mL), at a flow rate of 700 nL/min. A micro-fraction collector was used to deposit 1 mm spots every 5 s, and a total of 1408 fractions were collected in a 44×32 array for each NanoLC run. The column was washed with a gradient (0–100–100% B for 5 min and 2 min, respectively, at a flow rate of 800 nL/min) and equilibrated to 0% B for 3.5 min before subsequent injections. The fractionated samples were analyzed by a TOF/TOF 5800 System (SCIEX, Framingham, MA, USA) operated in positive ion reflector mode. The MALDI stage was set to continuous motion mode. MS data were acquired at 2400 laser intensity with 1000 laser shots/spectrum (200 laser shots/sub-spectrum) and MS/MS data were acquired at 3300 laser intensity with a DynamicExit algorithm and a high spectral quality threshold or a maximum of 1000 laser shots/spectrum (200 laser shots/sub-spectrum). Up to 20 top precursors with S/N > 40 in the mass range 750–4000 Da were selected from each spot for MS/MS analysis.

#### 5.4.4. Protein Identification

The resulting mass spectra were processed using ProteinPilot™ software v.5.0 (SCIEX, Framingham, MA, USA) and searched against a library of translated ORFs extracted from the *K. alborostrata* transcriptome. The Paragon 5.0.1.0 algorithm [79] embedded in ProteinPilot™ software (https://sciex.com/products/software/proteinpilot-software, accessed on 25 October 2021) was used in thorough mode with biological modifications and substitutions enabled. Carbamidomethyl on cysteine was specified as a fixed modification. The database also incorporated a list of common contaminants. Protein identifications with at least 95% confidence as determined by ProteinPilot™ software were considered significant. Proteins identified at least in two of nine samples were included in the final list.

Proteomic data are available in Supplementary File S5.

### 5.5. Mucus Toxins Analysis

For proteins identified in the mucus of *K. alborostrata*, signal peptides were predicted using SignalP v.5.0 [80]. Mature mucus peptides were predicted using the ConoPrec tool implemented in ConoServer [81]. Among mucus proteins, which were not automatically annotated, CRPs were searched manually based on a signal peptide and at least six cysteine residues. The 3D structures of CRPs were modeled and compared to known structures in the protein data bank using the protein homology/analogy recognition engine Phyre2 [82]. Functional annotation of CRPs was performed using CSPred v. 1.1 [62].

### 5.6. RT-qPCR

The relative expression of the three putative CRPs toxins, identified in the mucus of *K. alborostrata*, was evaluated in different parts of the body of the worm by a qRT-PCR. For the analysis nine specimens of *K. alborostrata* (six males and three females) were used. Individual specimens were relaxed in 7% MgCl2, fixed in RNALater solution, and dissected. The following parts were taken for the analysis and fixed in RNALater: head (including precerebral region before mouth), fragments of integument behind the mouth, proboscis, intestines (including body wall musculature), and gonads (ovaries with unfertilized eggs). Three biological replicates of each body part were obtained. Each replicate was pooled from three *K. alborostrata* specimens, separately for males and females. Total RNA was extracted similarly to in Section 5.3.1. Reverse transcription (RT) was performed on 2 μg of total RNA, treated with RNAse-free DNAse I (New England Biolabs, Ipswich, MA, USA), using the MMLV RT kit (Eurogen, Moscow, Russia). RT reactions were performed in the presence of 1 U/μL RNase Inhibitor (New England Biolabs, Ipswich, MA, USA). Samples were incubated for 60 min at 42 °C, 10 min at 70 °C, and cooled at 4 °C or stored at −80 °C. Glyceraldehyde-3-phosphate dehydrogenase (GAPDH), Actin-1 (act-1a), and 60S ribosomal protein L32 (Rp49) were chosen as reference genes for normalization of qPCR data. The primers were designed using Primer-BLAST tool [83], based on the mRNA sequences found in the transcriptome (Table 3). The primers were synthesized by Eurogen (Moscow, Russia). RT-qPCR was performed using 5X qPCRmix-HS SYBR Master Mix (Eurogen, Moscow, Russia) on CFX96 Touch Real-Time PCR Detection System (Bio-Rad, Hercules, CA, USA). The amplification procedure was as follows: 95 °C for 2 min for pre-mutability, and 42 cycles of 95 °C for 15 s, 54 °C for 15 s, and 72 °C for 15 s. Melting curve protocol was performed in the range of 65 °C to 95 °C, 0.5 °C per 5 s increment. Each biological replicate was analyzed in technical triplicate. The expression levels were calculated using the 2^−∆∆Ct^ method [84] with Rp49 as the reference gene. Calibration of expression levels was performed using the proboscis.

## Figures and Tables

**Table 1:** Annotated toxin candidates identified in the mucus proteome of *Kulikovia alborostrata*.

Protein	UniProt Accession	Transcript ID	*E*-Value	Whole Seq. Length	Mature Peptide Length	Seq. Coverage (%)	Mature Peptide Coverage (%)
Neurotoxins							
Nemertide alpha-1	P0DM24	ORF|076246	2.00 × 10^−5^	65	30	96%	76%
U1-nemetoxin-Csp1a	P60976	ORF|006024	1.00 × 10^−5^	69	27	95%	44%
Protease inhibitors							
U-actitoxin-Avd3I	P0DN10	ORF|006816	9.00 × 10^−18^	1352	1334	3%	-
Antistasin	P38977	ORF|016168	4.00 × 10^−6^	484	467	11%	-
Enzyme							
Astacin	P07584	ORF|006949	2.00 × 10^−28^	503	467	26%	-

**Table 2:** Functional classification of peptides according to CSPred v. 1.1.

Transcript ID	Probability Score				
	Ion Channel Blocker	Antimicrobial Peptide	Acetylcholine Receptor Inhibitor	Serine Protease Inhibitor	Hemolytic Peptide
ORF|076246	0.96	0.16	0.09	0.34	0.03
ORF|006024	0.81	0.06	0.03	0.23	0.1
ORF|001200	0.93	0.45	0.02	0.22	0.03

**Table 3:** Primer sequences used for RT-qPCR and reaction efficiency.

Gene	Forward Primer 5′-3′	Reverse Primer 5′-3′	Amplicon
*K.alb_GAPDH*	CGGCTACACTGAAGATAAGG	CCAACTTCGTTGTCATACCA	135 bp
*K.alb_act-1a*	TCATCAGGGTGTCATGGT	AGGATACCTCTCTTGCTCTG	78 bp
*K.alb_Rp49*	CCTCGTACACAATGTTAGGG	GCATTAGGATTGGTGACTTTG	150 bp
*K.alb_006024*	TCCGTGAATAAGAGATGCAG	CCTCGAGCATTCCTTGTATT	99 bp
*K.alb_001200*	TTTTCAAGAGGTGAAGGATGT	TAGATAGGCTGCTTGGGATT	98 bp
*K.alb_076246*	CAAGAGATGCAACCCAAAAG	TGTACATTTAAAGGCCCAGC	91 bp

## Data Availability

Raw transcriptomic data are available at NCBI’s GenBank under Bioproject PRJNA1190494 (accession numbers SRR31507531, SRR31507530, SRR31507529, SRR31507528).

## Supplementary Materials

The following supporting information can be downloaded at: https://www.mdpi.com/article/10.3390/toxins17010005/s1. Supplementary Table S1: Putative toxins in the transcriptome of *Kulikovia alborostrata*. Supplementary Table S2: Putative toxins in the proteome of *Kulikovia alborostrata*. Supplementary Table S3: Putative proteinaceous toxins identified in the phylum Nemertea. Supplementary File S1: *Kulikovia alborostrata* transcriptome assembly and annotation. Supplementary File S2: SwissProt, ToxProt, and nemertean toxin databases used in the analysis. Supplementary File S3: Alignments of putative toxin protein sequences identified in the mucus proteome of *Kulikovia alborostrata*. Supplementary File S4: Phyre2-based tertiary structure models of CRPs peptides (ORF|076246, ORF|006024, ORF|001200) found in the mucus of *Kulikovia alborostrata*. Supplementary File S5: Proteomic data of mucus of *Kulikovia alborostrata*.
